# Neuroendocrine responses to early life stress and trauma and susceptibility to disease

**DOI:** 10.1080/20008198.2017.1351218

**Published:** 2017-09-29

**Authors:** Panagiota Pervanidou, Agorastos Agorastos, Gerasimos Kolaitis, George P Chrousos

**Affiliations:** ^a^ Unit of Developmental & Behavioral Pediatrics, First Department of Pediatrics, Medical School, National and Kapodistrian University of Athens, “Aghia Sophia” Children’s Hospital, Athens, Greece; ^b^ Department of Psychiatry and Psychotherapy, University Medical Center Hamburg-Eppendorf, Hamburg, Germany; ^c^ Department of Child Psychiatry, Medical School, National and Kapodistrian University of Athens, “Aghia Sophia” Children’s Hospital, Athens, Greece

**Keywords:** Early life stress, stress response, stress hormones, post-traumatic stress disorder (P.T.S.D.), physical health, mental health

## Abstract

The term ‘early life stress’ has been used to describe a broad spectrum of adverse exposures during foetal life, childhood and adolescence. Early life stress and trauma are associated with a higher risk for later mental and physical health disorders, such as anxiety, depression, and post-traumatic stress disorder (P.T.S.D.) as well as cardiometabolic and inflammatory diseases and chronic pain syndromes. The objective of this brief review is to investigate the neuroendocrine responses to early life stress and their role as biological predisposing factors for later disease.

Stress-related neuroendocrine alterations in response to early adversity include hyper- or hypo-activation of the stress system and may persist or worsen in later life, acting as biological vulnerability factors for the development of later disease. A key effect of stress during foetal life, childhood, and adolescence is that it programmes the developing brain, especially brain structures involved in stress reactions, such as the prefrontal cortex, the hippocampus, and the amygdala, to hyper- or hypo-react to ensuing stressors. Animal studies have shown that chronically elevated stress mediators may lead to alterations in brain development through mechanisms of accelerated loss of neurons, delays in myelination, or abnormalities in developmentally appropriate neural synaptic pruning. Critical periods of brain development represent time-windows of elevated synaptic plasticity, mediating vulnerability, or establishing resilience to stress.

Stress is generally associated with acute activation of the hypothalamic-pituitary-adrenal (H.P.A.) axis and the arousal/sympathetic nervous system, as evidenced, in most studies, by elevated cortisol and catecholamine concentrations in the periphery. However, the chronic and/or intense experience of stress may be associated with chronic hyper- or hypo-activation of mediators of the stress system. This chronic condition represents dyshomeostasis, also called allostasis or cacostasis, which is related to further morbidity, such as obesity and the metabolic syndrome, diabetes mellitus type 2, atherosclerosis, osteoporosis, and immune dysfunction (Pervanidou & Chrousos, 2012).

P.T.S.D., the most common stress-related disorder, is typically associated with increased secretion of corticotropin-releasing hormone centrally, with paradoxically decreased cortisol secretion peripherally, and elevated circulating catecholamine concentrations. Stress-related neuroendocrine alterations during early life might result in conditions characterized by chronic hypo-activation of the H.P.A. axis, as typically observed in P.T.S.D. and atypical depression. Scarce data exist on the longitudinal course of P.T.S.D. development and maintenance, beginning from the exposure to the traumatic event. We investigated the natural history of neuroendocrine changes in relation to P.T.S.D. development in children and adolescents experiencing a motor vehicle accident. This longitudinal study provided evidence for an initial elevation of evening salivary cortisol and an alteration in cortisol circadian secretion in response to the experience of an intense stressor. This initial alteration was followed by a gradual normalization of cortisol with time, leading potentially to decreased cortisol concentrations in the periphery, months or years after the traumatic exposure. At the same time, a progressive elevation of circulating norepinephrine (N.E.) was noted in children that continued to exhibit P.T.S.D. This longitudinal interaction of peripheral cortisol and NE concentrations seems to characterize those that develop and maintain P.T.S.D. (Pervanidou et al., 2007). Thus, low cortisol, together with high N.E. concentrations, that characterize adult P.T.S.D., may be the end stage of the disorder. These data are also in accordance with findings in adults, showing hypocortisolism in the aftermath of a stressful experience to predict P.T.S.D. development.

Several parameters affect the direction of the H.P.A. axis (increased or decreased cortisol production) in P.T.S.D., and among them, previous trauma and time since the trauma occurred seem to be crucial determinants of hyper- or hypo- activation of the H.P.A. axis. Other parameters include: genetic and epigenetic vulnerability; transgenerational actions; environmental influences in the management of early life stress; the nature, severity, and duration of the traumatic event; the developmental stage, age at the time of trauma; and comorbidities (Pervanidou, 2008).

In conclusion, early life stress exposures result in neuroendocrine alterations and programming of the neuronal networks of the brain, acting as predisposing factors for further mental and physical health problems.
